# Supplementary material for: Hydrogen Bonds under Stress: Strain-Induced Structural Changes in Polyurethane Revealed by Rheological Two-Dimensional Infrared Spectroscopy
Source: J Phys Chem Lett. 2023 Jan 23;14(4):940–6. doi: 10.1021/acs.jpclett.2c03109 (PMC9900637; doi:10.1021/acs.jpclett.2c03109)
Supplement: Supplementary file 1 — jz2c03109_si_001.pdf [file jz2c03109_si_001.pdf]

Supplementary for: Hydrogen Bonds under Stress: Strain-Induced Structural Changes in Polyurethane revealed by Rheological Two-Dimensional Spectroscopy

Giulia Giubertoni,[*,†] Michiel Hilbers,[†] Federico Caporaletti,[‡,†] Peter Laity,[¶] Hajo Groen,[†] Anne Van der Weide,[†] Daniel Bonn,[‡] and Sander Woutersen[*,†]

†*Van 't Hoff Institute for Molecular Sciences, University of Amsterdam, Science Park 904, 1098XH Amsterdam, The Netherlands*

‡*Van der Waals-Zeeman Institute, Institute of Physics, University of Amsterdam, 1098XH Amsterdam, The Netherlands*

¶*Department of Materials Science and Engineering, University of Sheffield, Sir Robert Hadfield Building, Mappin St., Sheffield S1 3JD, UK*

E-mail: g.giubertoni@uva.nl; s.woutersen@uva.nl

Sample characterization

Samples were obtained by cutting condom in pieces of an area of 1 cm^2. To characterize the soft segments, we study the infrared region around 1100 cm^{-1}, also called finger-print region. We observe a strong band at 1110 cm^{-1}, which is assigned in literature to a stretching mode of CH_2-O-CH_2 of polyether, suggesting that the soft segments are made by polyether. This is confirmed by the fact we do not observe any strong band at 1180 cm^{-1} that is assigned to C-O-C mode in polyester. For band assignement, see ref.[1]

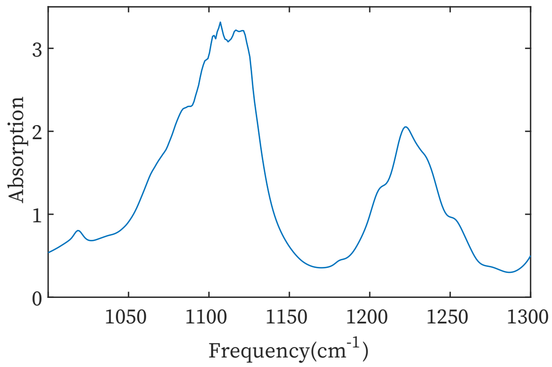

Figure S1: IR spectrum of a sample taken from Sagami 001 at zeros stress condition.

Nodal line slope Protex 002

We perform rheo-2DIR on sample taken from Protex 002. Because Protex 002 is thicker, the absorption of the carbonyl peaks is higher with respect to the Sagami 001, and thus at zero-stress condition most of the IR light is being absorbed by the sample. Due to this, we can perform rheo-2DIR just when the sample is already deformed, and thus thinner. In Fig.S6 we report the data and analysis of a sample subjected to three consecutive stress cycles up to 600 %. As reported in the main text, we again observe a decrease of the NLS upon recovery of the lowest possible deformation and a shift toward lower frequency of the bleach signal (Fig.S6 C-D).

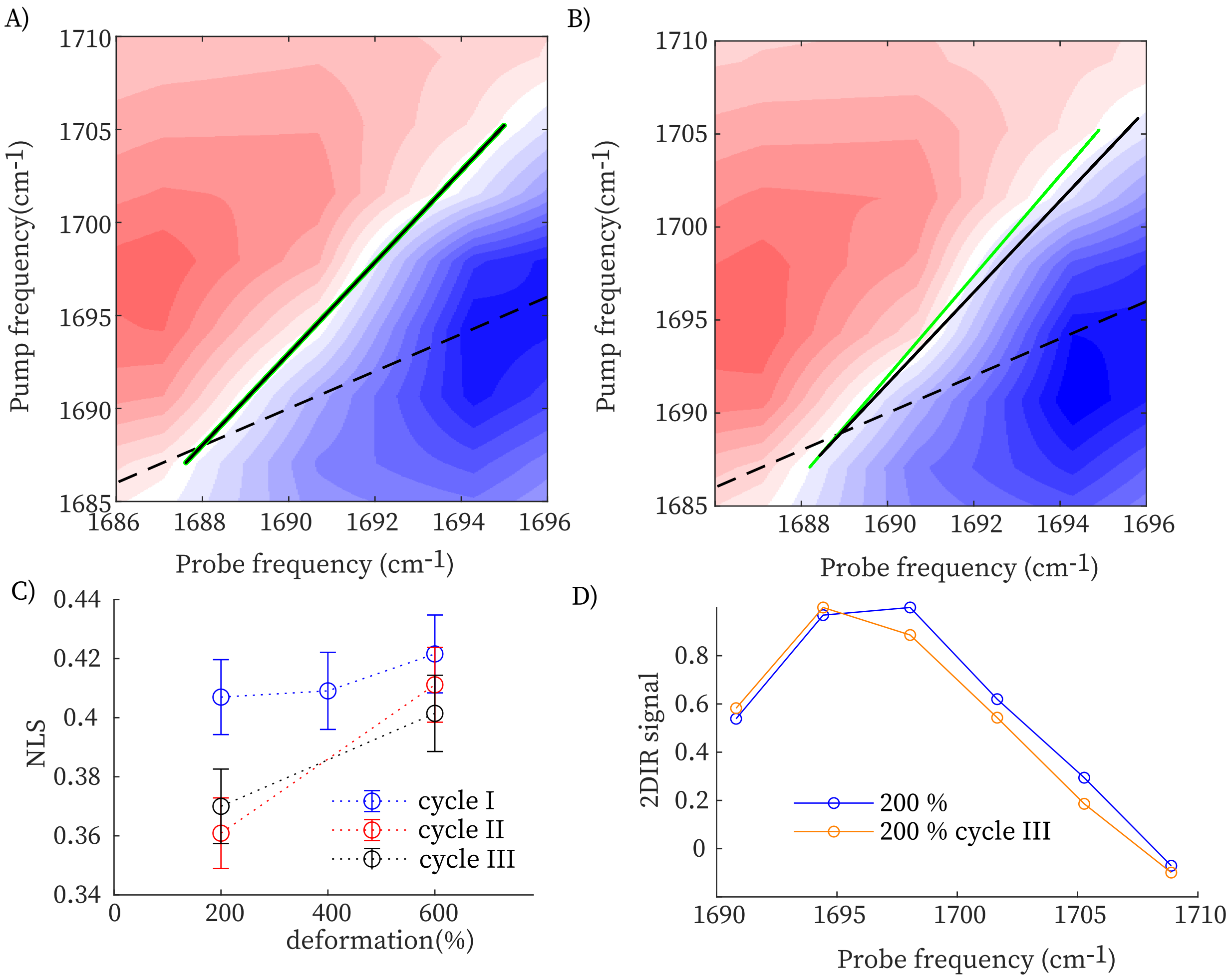

Figure S2: A-B) 2DIR spectra and nodal line slopes of a sample at 200 % of deformation and upon recovery of 200 % stress condition after a deformation up to 600%, respectively. C) NLS slopes for three subsequent cycles. D) Comparison of bleach diagonal slices extracted by 2DIR spectra in A-B.

Nodal line slope Protex 002 subjected to deformation at high temperature

Samples were deformed at high temperature to reduce the thickness from 30 to 25 μm. By doing so, we observed that the NLS starts from a lower value with respect to the untreated one, and increases during the cycle.

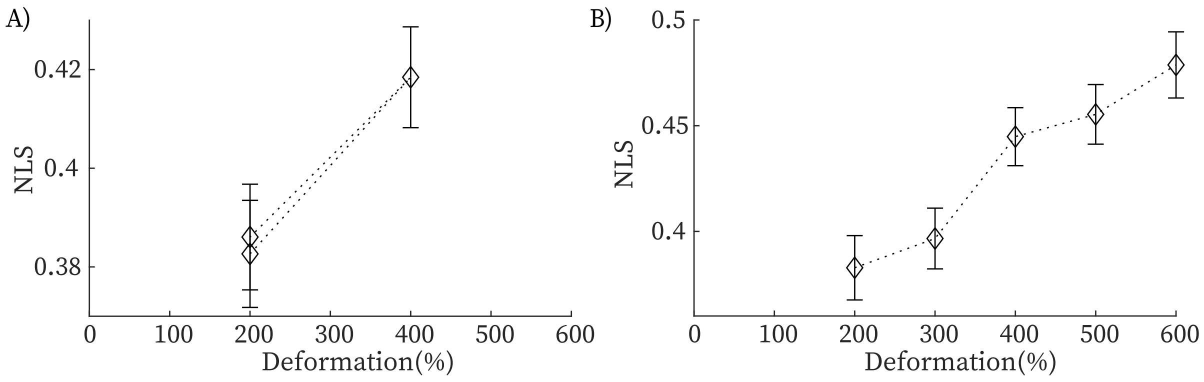

Figure S3: A-B) Nodal line slope of a sample from Protex 002 that was deformed at high temperature (70-100 °C) for two different experiments cycling until 400 % and 600 % respectively.

Effect of cleaning procedure on the NLS

To check whether cleaning with ethanol influences the nodal line slope, we removed the lubricant from the sample without using ethanol, by simply wiping the condom with a clean tissue (Fig.S4). The removal of the lubricant was confirmed by the disappearance of the 1260 cm-1 band of the silicone oil.[2] We then measure the NLS at zero stress and at zero-stress recovery after 500% deformation (Fig.S4) at different time delays. The results show that the decrease in the NLS upon deformation is observed independently from the cleaning procedure that was used. Comparing the slopes obtained in this experiment (Sagami 001, III) with the ones obtained on samples cleaned with ethanol (Sagami 001, I and Sagami 001, II), we observe that there is no significant difference between the NLS slope (also confirmed for the CLS in Fig.S7), indicating that the ethanol does not affect the HB distribution (Fig.S7).

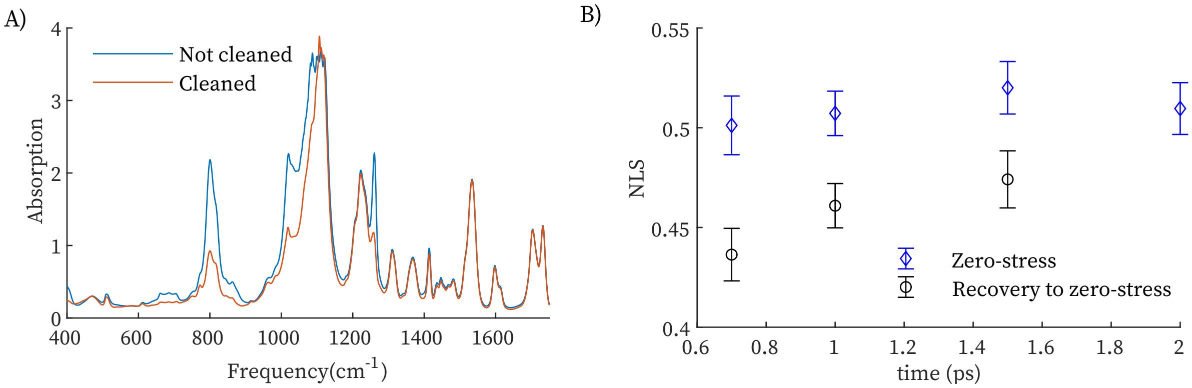

Figure S4: A) IR spectra before and after cleaning the condom with a soft tissue. B) NLS for the same sample at different time delays.

Infrared spectra Sagami 001 at different deformations

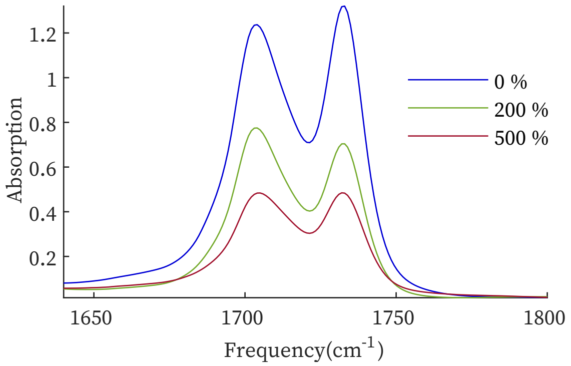

Figure S5: Infrared spectra of Sagami 001 at different deformations without baseline correction.

Infrared spectra Sagami 001 and Protex 002

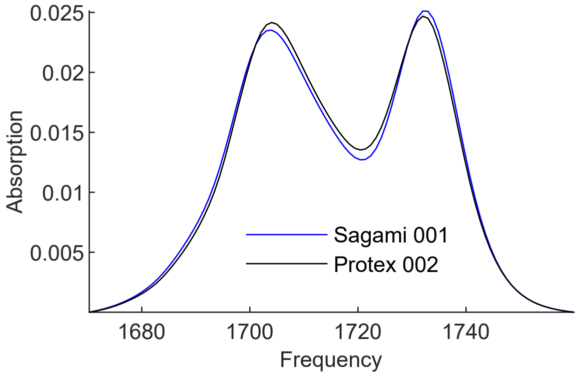

Figure S6: Comparison between the infrared spectra of Sagami 001 and Protex 002 at zero-stress condition.

Central line slope

We calculated the center line slopes of the bleach for different experiments on the Sagami 001 and Protex 002 (Fig.S7). To do so, we first fit a series of cuts through the 2D spectrum that are parallel to the probe frequency axis by using two Lorentzian-shaped peaks that describe the bleach and the ESA. We then fit a line through the center positions of the bleach obtained from the fit of the cuts. We find that the CLS is 0.50 ± 0.02 for the zero-stress and 0.38 ± 0.02 for recovery at zero-stress conditions, where the values and errors represent the mean and the standard deviations obtained over 4 measurements.

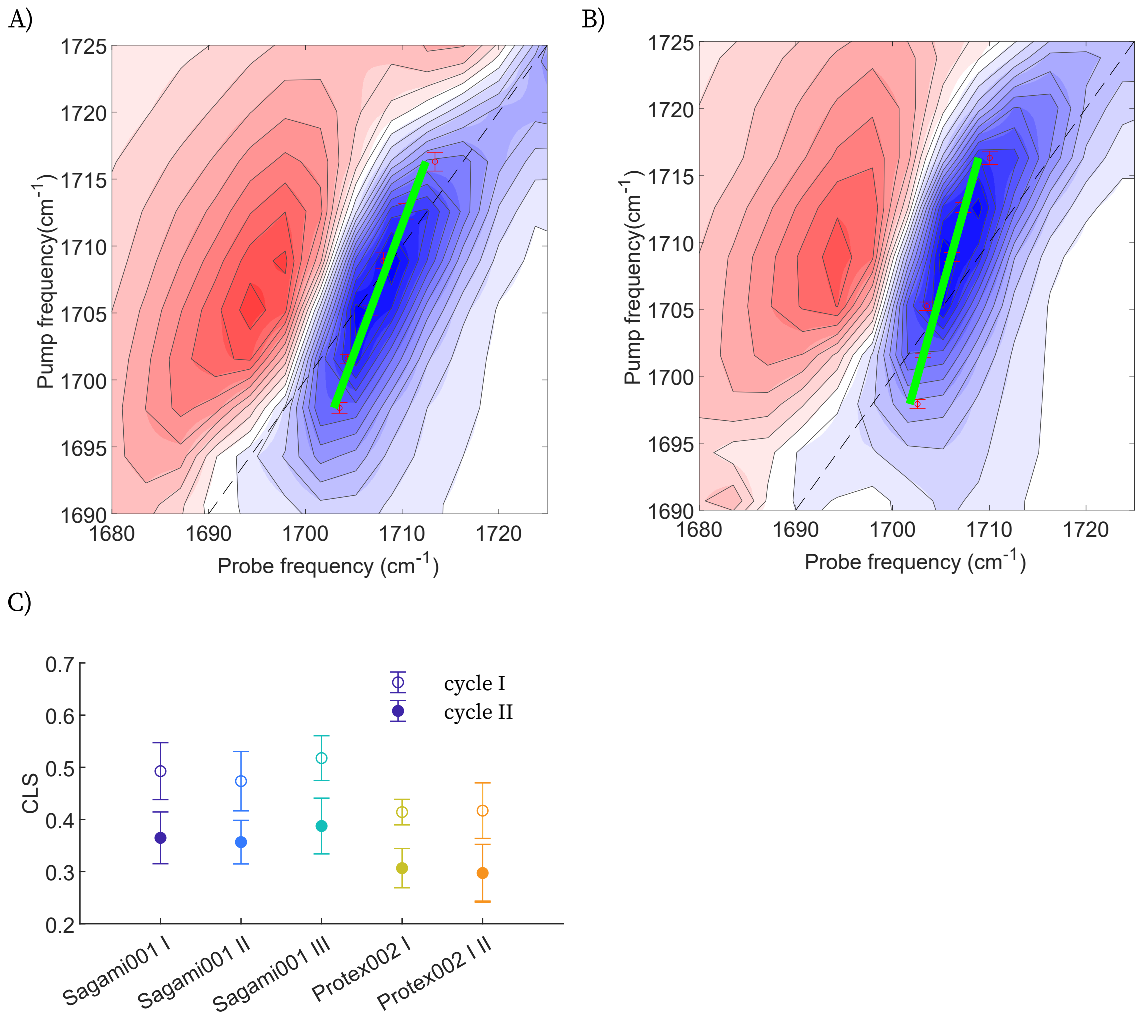

Figure S7: A) and B) 2DIR spectra and central lines for Sagami 001 at zero-stress and at recovery zero-stress condition after a deformation cycle, respectively. C) central line slope values calculated for the Sagami 001 and Protex 002.

References

(1) Shibayama, M.; Kawauchi, T.; Kotani, T.; Nomura, S.; Matsuda, T. Structure and Properties of Fatigued Segmented Poly(urethaneurea) I. Segment Orientation Mechanism due to Fatigue. *Polymer Journal 1986 18:10* **1986**, *18*, 719–733.

(2) Mandrile, L.; Giovannozzi, A. M.; Pennecchi, F.; Saverino, A.; Lobascio, C.; Rossi, A. M.

Direct detection and quantification of molecular surface contaminants by infrared and Raman spectroscopy. *Anal. Methods* **2015**, *7*, 2813–2821.
